# Case Report: Synergistic potential of RANKL and ACLY inhibition in a breast cancer patient with acquired resistance to CDK4/6 inhibitors

**DOI:** 10.3389/fonc.2026.1911207

**Published:** 2026-08-26

**Authors:** Anastasiia Samusieva, Ielizaveta Gorodetska, Oleg Socha, Yuliia Lytovchenko, Roman Kisielov, Borys Dons`koi, Daria Kozeretska, Vasyl Lukiyanchuk, Anna Dubrovska, Valeriy Cheshuk, Olga Ponomarova, Iryna Kozeretska

**Affiliations:** 1 Department of Oncology, Shupyk National Healthcare University of Ukraine, Kyiv, Ukraine; 2 OncoRay-National Center for Radiation Research in Oncology, Faculty of Medicine and University Hospital Carl Gustav Carus, Technische Universität Dresden and Helmholtz-Zentrum Dresden-Rossendorf, Dresden, Germany; 3 Institute of Radiooncology-OncoRay, Helmholtz-Zentrum Dresden-Rossendorf (HZDR) Dresden, Dresden, Germany; 4 Provincial Hospital. St. Padre Pio, Przemy´sl, Poland; 5 Radiology Department, Kyiv City Clinical Oncology Center, Kyiv, Ukraine; 6 Department of Radiodiagnostics, Shupyk National Healthcare University of Ukraine, Kyiv, Ukraine; 7 ”Victor Chernyshov" Laboratory of Immunology, State Institution "Ukrainian Center of Maternity and Childhood", National Academy of Medical Sciences of Ukraine, Kyiv, Ukraine; 8 Independent Consultant, Kyiv, Ukraine; 9 Institute of Radiooncology - OncoRay, Helmholtz-Zentrum Dresden - Rossendorf (HZDR), Dresden, Germany; 10 German Cancer Consortium (DKTK), Partner Site Dresden, Dresden, Germany; 11 German Cancer Research Center (DKFZ), Heidelberg, Germany; 12 National Center for Tumor Diseases (NCT), Partner Site Dresden, Dresden, Germany; 13 Department of Oncology, Bogomolets National Medical University, Kyiv, Ukraine; 14 Department of Chemotherapy #1, Kyiv City Clinical Oncology Center, Kyiv, Ukraine; 15 Department of General and Molecular Genetics, National Taras Shevchenko University, Kyiv, Ukraine

**Keywords:** breast cancer, CDK4/6 inhibitor resistance, lymph node metastasis, bone metastasis, RANKL inhibition, LGR4, ATP-citrate lyase, bempedoic acid

## Abstract

**Background:**

The development of acquired resistance to cyclin-dependent kinase 4/6 inhibitors (CDK4/6i) represents a major clinical challenge in the management of hormone receptor-positive (HR+), human epidermal growth factor receptor 2 (HER2)-negative metastatic breast cancer. Overcoming this resistance requires novel therapeutic strategies that target the underlying molecular escape pathways.

**Case presentation:**

We report the case of a patient initially diagnosed in 2011 with pT2N0M0, ER+/HER2- invasive ductal carcinoma. Following multimodality treatment, she experienced disease progression with bone metastases after five years, followed by lymph node metastases at the ten-year mark. Subsequent treatment with the CDK4/6i ribociclib in combination with fulvestrant resulted in disease progression approximately one year later, indicating acquired resistance. Due to progressive bone loss, denosumab was initiated for skeletal support, and bempedoic acid was later added for dyslipidemia.

**Discussion:**

This case provides a clinical basis for exploring two innovative therapeutic concepts to overcome resistance. First, we discuss the complex role of receptor activator of nuclear factor kappa-B ligand (RANKL) inhibition. Given that leucine-rich repeat-containing G protein-coupled receptor 4 (LGR4) functions as a decoy receptor for RANKL, we hypothesize that a patient’s LGR4 expression status may dictate the efficacy and safety of denosumab, potentially serving as a predictive biomarker to personalize its use. Second, we propose that bempedoic acid, a supportive care medication, may exert a direct anti-neoplastic effect. Its dual mechanism, inhibiting ATP-citrate lyase (ACLY) to disrupt lipid synthesis and activating AMP-activated protein kinase (AMPK) to suppress mammalian target of rapamycin complex 1 (mTORC1) signaling, positions it as a potential agent to counteract the metabolic reprogramming associated with therapeutic resistance.

**Conclusion:**

This case report illustrates the sequential development of acquired therapeutic resistance in the long-term management of metastatic breast cancer. Although a single clinical observation cannot establish definitive efficacy, this scenario generates compelling hypotheses for future studies. It highlights the potential need to explore biomarker-driven approaches—such as evaluating LGR4 expression to guide RANKL inhibition—to personalize treatment. Additionally, it raises the possibility that repurposed supportive care medications, such as bempedoic acid, might offer hypothetical anti-neoplastic benefits, warranting broader clinical validation to overcome complex resistance mechanisms.

## Introduction

Breast cancer (BС) remains the most frequently diagnosed malignancy among women worldwide and the leading cause of cancer-related mortality in the female population in many countries, including Ukraine (1). According to recent global statistics, over 2.3 million new cases are diagnosed annually, representing nearly one in four of all cancers in women (2). The most common molecular subtype of breast cancer is Luminal A. This subtype is part of the larger hormone receptor-positive (HR+), human epidermal growth factor receptor 2 (HER2)-negative category, which accounts for approximately 65–70% (3).

**Figure 1:**
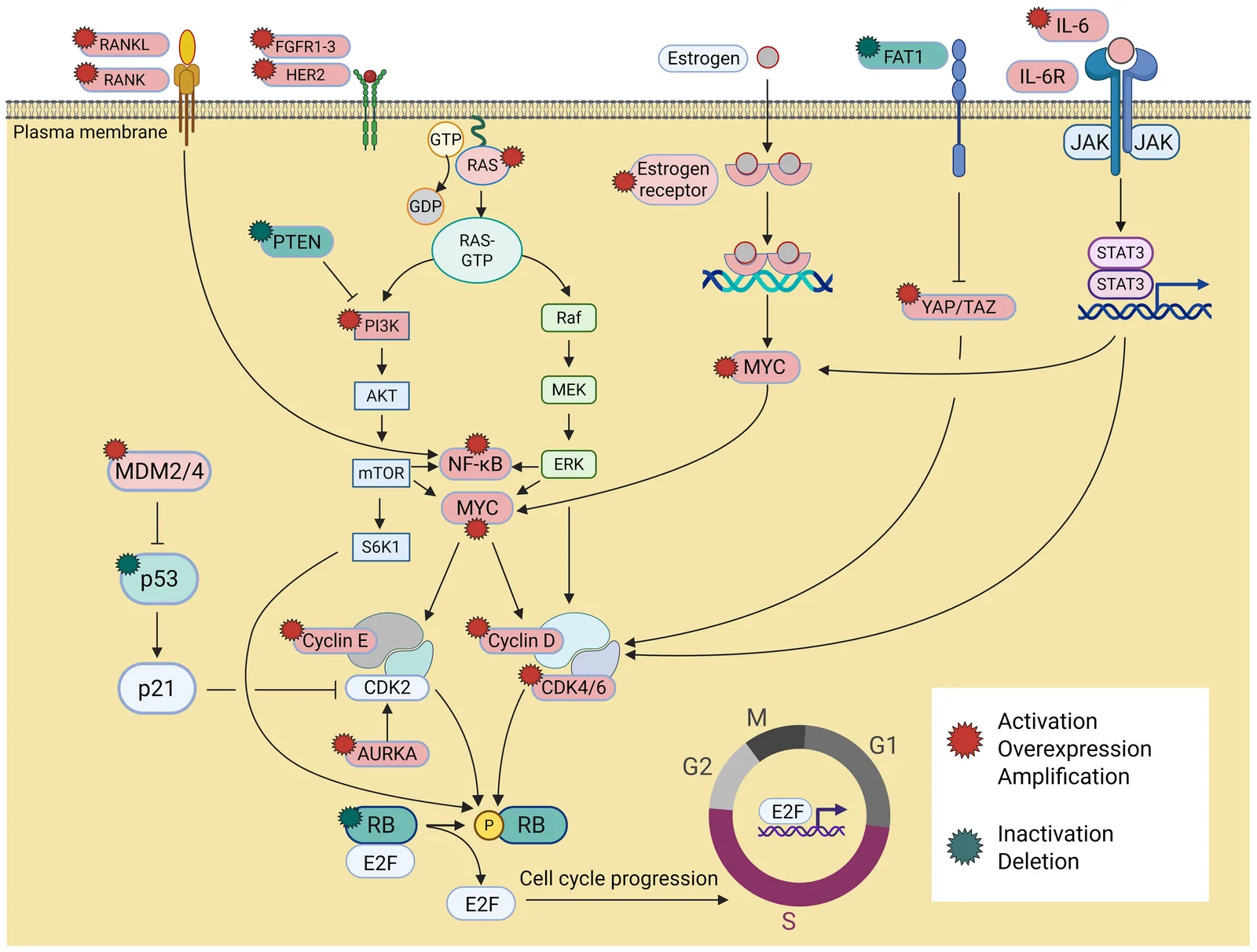
Schematic representation of selected resistance mechanisms and biomarkers of resistance to CDK 4/6 inhibitors in combination with endocrine therapy. Created in BioRender. Dubrovska, A. (2026). AKT, Protein Kinase B; AURKA, Aurora Kinase; CDK2, Cyclin-Dependent Kinase 2; CDK4/6, Cyclin-Dependent Kinase 4/6; E2F, E2 transcription Factor; ERK, Extracellular signal-Regulated Kinase; FAT1, FAT atypical cadherin 1; FGFR1-3, Fibroblast Growth Factor Receptor 1-3; G1, S, G2, M, Gap 1, Synthesis, Gap 2, Mitosis; GDP, Guanosine diphosphate; GTP, Guanosine-5’-triphosphate; HER2, Human Epidermal growth factor Receptor 2; IL-6, Interleukin 6; IL-6R, Interleukin 6 Receptor; JAK, Janus Kinase; MDM2/4, Mouse double minute 2/4 homolog; MEK, Mitogen-activated protein kinase; mTOR, Mammalian Target of Rapamycin; MYC, Master Regulator of Cell Cycle Entry and Proliferative Metabolism; NF-κB, Nuclear Factor kappa-light-chain-enhancer of activated B cells; p21, Cyclin-dependent kinase inhibitor 1; p53, Tumor protein p53; PI3K, Phosphoinositide 3-Kinase; PTEN, Phosphatase and Tensin homolog; Raf, Rapidly Accelerated Fibrosarcoma; RANK, Receptor Activator of Nuclear factor Kappa-B; RANKL, Receptor Activator of Nuclear factor Kappa-B Ligand; RAS, Rat Sarcoma virus homolog; RB, Retinoblastoma protein; S6K1, Ribosomal protein S6 Kinase beta-1; STAT3, Signal Transducer and Activator of Transcription 3; YAP/TAZ, Yes-Associated Protein/Transcriptional coactivator with PDZ-binding motif.

The introduction of cyclin-dependent kinase 4/6 inhibitors (CDK4/6i) has significantly transformed the therapeutic landscape for HR+/HER2− advanced breast cancer. By selectively inhibiting cell cycle progression from the G1 to S phase, these agents enhance the efficacy of endocrine therapy, prolong progression-free survival, and improve overall outcomes (4). Nevertheless, the clinical success of CDK4/6i has been paralleled by the rapid characterization of diverse mechanisms of acquired resistance, and the number of documented escape pathways continues to grow (**Figure 1**) (5–7).

## Case presentation

The 47-year-old patient K. had a history of invasive carcinoma of the right breast diagnosed and treated in 2011. As the first therapy line, subcutaneous nipple-sparing mastectomy with regional lymphadenectomy and immediate breast reconstruction with silicone breast implant (POLYTECH Health & Aesthetics, Germany) was performed. Pathologic report of postoperative material demonstrated intermediate grade (G2) invasive ductal carcinoma with negative margins and negative lymph nodes (pT2N0M0). The immunohistochemical report: ER – 10%, PR – 68%, HER2/neu – negative, Ki67 – 50%. Surgical treatment was followed by adjuvant chemotherapy (6 courses of fluorouracil + doxorubicin + cyclophosphamide) and hormonotherapy (tamoxifen 20mg/day for 5 years (from 2011 to 2016) with Zoladex^®^ (AstraZeneca, Great Britain and Switzerland) 3,6 mg/every 28 days for 5 years (from 2011 to 2016). In December 2016, a follow-up examination revealed metastasis in the sternum bone, and the patient underwent a course of radiation therapy at this zone (total dose 20 Gy) and zoledronic acid 4mg every 4 weeks for 2 years. In February 2017, the patient underwent oophorectomy.

In 2021, a second progression was observed, with foci in the right supraclavicular node, the sternum, and the lymph nodes of the left axillary region. Next, the patient was included in a clinical trial, SERENA-2 (8), since February 2021. In this study, the patient received therapy with the investigational agent, AZD9833, and achieved disease stabilization by the fall of 2023. Subsequently, due to the imposition of martial law in her home country, she continued participation in the same clinical trial at one of the collaborating research centers in Poland (Rzheshuw, Mrukmed).

Since July 2023, two more lymph nodes have been enlarged during follow-up examinations. This results in termination of participation in the clinical trial. Since December 2023, the patient has been prescribed the CDK4/6 inhibitor ribociclib 600 mg orally, once daily for 21 consecutive days, followed by a 7-day break and fulvestrant (Faslodex) 500mg every 4 weeks. During the first 3 months of the course, a 20 percent reduction in nodes was noted by CT, and during the next 3 months, no further progress was observed. In the next 6 months, a gradual increase in size was noted on CT scans. After assessing the condition of the skeletal system, local bone loss in the vertebrae was detected (**Supplementary Figure 1**).

In October 2024, denosumab was initiated at a dose of 60 mg (Prolia) specifically for management of treatment-induced bone loss. Subsequently, the dosing regimen was escalated to 120 mg (Xgeva). The specific clinical indication for this dose adjustment in our patient was a deliberate strategy to maximize nuclear factor kappa-B ligand (RANKL) inhibition due to suspected resistance. Based on the literature, the patient’s serum RANKL protein level was assessed during denosumab use in combination with ribociclib (9, 10).

In March 2025, the patient’s lipid profile showed a trend toward increasing low-density lipoproteins (2.74 mmol/L), and she was prescribed bempedoic acid orally, 1 tablet/day (Nilemdo, 180 mg per tablet).

Considering the patient’s disease course, metastatic lymph nodes in the right supraclavicular region were selected as marker foci. Two methods of monitoring dynamics were chosen to provide both frequent and comprehensive assessments of tumor burden: (i) high-frequency monitoring performed by ultrasound examinations once every three weeks to detect subtle changes in the size and morphology of the marker lymph nodes, (ii) comprehensive staging performed by CT scans with contrast enhancement was conducted once every three months for a broader assessment of the overall disease status. This dual-monitoring strategy (graphically represented in **Supplementary Figures 2**–**17**) provides a detailed timeline of the patient’s response and eventual progression on ribociclib, documenting the clinical development of resistance.

The patient’s comorbidities included a history of viral hepatitis А (1970), bronchial asthma diagnosed in 2013, and hypothyroidism from 2010.

## Discussion

This case report describes the long-term management of a patient with ER-positive, HER2-negative metastatic breast cancer over a period of more than a decade. Overall, the case highlights the sequential nature of treating advanced breast cancer, the development of resistance to various therapies over the treatment course, and the importance of managing both the cancer and the side effects of its long-term treatment.

Denosumab is a human monoclonal IgG2 antibody that targets the receptor activator of RANKL, which binds to RANK with high affinity and specificity, preventing the interaction between RANKL and RANK. RANK is involved in all steps of breast tumor development: from initial tumor formation to cancer cell migration, and subsequent metastasis formation (11). It was demonstrated that RANK overexpression in luminal BC is associated with intrinsic resistance to CDK4/6i, as shown in both *in vitro* and *in vivo* xenograft models (10). At the same time, results of subsequent studies demonstrate the clinical relevance of the combination of CDK4/6i and endocrine therapy (ET) with RANKL inhibition (9, 12).

Also, one recent publication indicates that increased RANKL expression is associated with a favorable prognosis in breast cancer patients with high levels of leucine-rich repeat-containing G protein-coupled receptor 4 (LGR4). LGR4 is a regulator of osteoclast formation and function. LGR4 appears to act as a negative regulator of RANKL-induced osteoclastogenesis, meaning that loss of LGR4 function can lead to increased osteoclast activity and subsequent bone loss, or osteoporosis (13). Inhibition of RANKL reduced breast cancer stem cell (BCSC) properties in LGR4-low cell lines, while promoting migration in LGR4-high cells. In addition, the RANKL/RANK axis activated NF-κB signaling and increased BCSC in RANK-overexpressing cells. In contrast, in cells overexpressing LGR4, RANKL did not activate NF-κB, but instead inhibited the Wnt/β-catenin pathway, leading to a decrease in BCSC numbers (14). Thus, the decision to downregulate RANKL expression with denasumab requires prior assessment of LGR4 expression in the individual patients.

In the EU, Nilemdo is a medicine for lowering levels of cholesterol in the blood and is used to reduce blood cholesterol levels in adults with primary hypercholesterolemia or mixed dyslipidemia (conditions that cause high levels of fats, including cholesterol, in the blood). Patients taking the medicine are required to be on a low-fat diet (15). At the same time, the literature indicates that bempedoic acid activates AMP-activated protein kinase (AMPK), which, in turn, phosphorylates and inhibits the protein Raptor, acting as a scaffold on which the components of the mammalian target of rapamycin complex 1 (mTORC1) are assembled (16–18). That is, if Raptor is blocked by AMPK, the efficiency of mTORC1 complex formation is significantly lower, and therefore its function in activating the cell cycle is reduced. ATP-citrate lyase (ACLY) is a crucial enzyme that links the metabolism of glucose to the synthesis of lipids. ACLY is often found at high levels in many types of cancer, including breast, lung, liver, and colorectal cancers, and its overexpression is linked to tumor growth (19). Therefore, ACLY inhibitors, such as bempedoic acid (ETC-1002), are being explored not only for their established role in lowering cholesterol but also as potential anti-cancer agents that can disrupt the metabolic engine of tumors (16, 20–22).

In recent years, a significant body of research has identified the IL-6/STAT3 signaling pathway as a critical driver of resistance to CDK4/6 inhibitors across different subtypes of breast cancer. This pathway plays a dual role in undermining therapy. In hormone receptor-positive breast cancer, activation of the IL-6/STAT3 axis is a key mechanism of acquired resistance, with circulating IL-6 levels rising in patients as their disease progresses (6, 23, 24).

Consequently, targeting this pathway has emerged as a promising therapeutic strategy. Preclinical studies show that combining CDK4/6 inhibitors with STAT3 inhibitors can re-sensitize resistant tumors to treatment and suppress metastasis (6). This approach not only counteracts acquired resistance but also uncouples the undesirable pro-tumor SASP effects from the beneficial cell cycle arrest induced by the therapy. Therefore, inhibiting the IL-6/STAT3 axis represents a transformative strategy to overcome resistance and enhance the efficacy of CDK4/6 inhibitors.

## Conclusion

In summary, this case report documents the clinical course of a heavily pretreated, CDK4/6i-resistant metastatic breast cancer. Following the co-administration of denosumab and bempedoic acid in March 2025, the patient achieved overall disease stabilization. Despite some mixed nodal dynamics at the last control examination—where the target lymph node measured 3.2 × 2.28 × 2.79 cm alongside other nodes (1.49 × 0.89 cm and 0.44 × 0.41 cm)—the maximum dimensions of the target lesion did not exceed its initial baseline size of 3.58 × 2.50 × 2.86 cm.

While the temporal association between this clinical stabilization and the introduction of these agents is compelling, we explicitly acknowledge the limitations inherent to a single-patient observation. Because the patient received concurrent therapies, direct causality cannot be definitively established.

Nevertheless, this clinical scenario generates two plausible, exploratory hypotheses for future research. First, we hypothesize that LGR4 expression might modulate the efficacy of RANKL inhibition, suggesting that the RANKL/LGR4 signaling axis could eventually serve as a predictive biomarker for personalized denosumab therapy. Second, the case raises the possibility that supportive care medications, specifically bempedoic acid, might possess anti-neoplastic properties via metabolic modulation (ACLY/AMPK/mTORC1) in a clinical setting.

The future of advanced breast cancer management relies on such nuanced, biomarker-driven approaches. However, rigorous molecular validation, standardized LGR4 assessment, and larger clinical cohort studies are essential to determine whether targeting these pathways can safely and effectively overcome acquired resistance to CDK4/6 inhibitors.

## Data Availability

The original contributions presented in the study are included in the article/**Supplementary Material**, further inquiries can be directed to the corresponding author/s.
