# Supplementary material for: Case Report: Synergistic potential of RANKL and ACLY inhibition in a breast cancer patient with acquired resistance to CDK4/6 inhibitors
Source: Front Oncol. 2026 Aug 26;16:1911207. doi: 10.3389/fonc.2026.1911207 (PMC13553352; doi:10.3389/fonc.2026.1911207)
Supplement: Supplementary file 1 [file Table1.docx]

*Disease Dynamics on Follow-Up Diagnostics*

The image demonstrates a CT scan of the lumbar spine in coronal and sagittal planes (Figure S1). The red arrows indicate degenerative–dystrophic changes of the lumbar spine, including:

- degenerative alterations of the intervertebral discs and adjacent vertebral bodies,

- decreased intervertebral space height,

- irregularity of the vertebral endplates,

- extra bony growths (osteophytes) along the margins of the vertebral bodies.

**Figure S1. The CT scan image of the lumbar spine in coronal and sagittal planes.**

Considering the patient's disease course, metastatic lymph nodes in the right supraclavicular region were selected as marker foci. Two methods of monitoring dynamics were chosen: an ultrasound (US) examination once every three weeks, and a CT with contrast once every three months.

Ultrasound reports:

At the initial presentation, a hypoechoic heterogeneous lymph node with well-defined contours and partially finely lobulated margins, showing a pronounced lobular echostructure, was identified in the right cervical–supraclavicular region. Its size was 3.58 × 2.50 × 2.86 cm (width × depth × length). The lymph node demonstrated signs of intimate adherence to the cervical vascular bundle without clear evidence of invasion (Figure S2).

**Figure S2. Initial US of the metastatic lymph node.**

Color Doppler mapping (CDC) shows prominent large vessels of intranodular neovascularization, Node-RADS 5 (Figure S3).

**Figure S3. Initial CDC of metastatic lymph node.**

After 3 weeks, the size decreased to 3.31×2.49×2.83 cm, with signs of single hyperechoic fibrous inclusions in the lower medial segment against the background of a heterogeneous lymph node structure (Figure S4).

**Figure S4. US of a metastatic lymph node after the first 3 weeks.**

At the next visit, a tendency towards a decrease in the node size of 3.31×2.38×2.82 cm and a change in the echo structure due to pronounced areas of alternating reduced and increased echogenicity were observed (Figure S5, 6).

**Figure S5. US of a metastatic lymph node with changes in echostructure due to pronounced areas of alternating reduced and increased echogenicity (1).**

**Figure S6. US of a metastatic lymph node with changes in echostructure due to pronounced areas of alternating reduced and increased echogenicity (2).**

Against this background, in the dynamics after a month, a hyperechoic area of fibrosis was registered in the lower medial segment of the neoplasm, 0.35×0.3 cm (Figure S7).

**Figure S7. US of a metastatic lymph node with a hyperechoic area of fibrosis in the inferomedial segment.**

There was a decrease in width to 3.23×2.44×2.88 cm. According to the control ultrasound study, the node continued to decrease to 3.19×2.43×2.86, and the fibrosis zone increased to 0.37×0.34 mm (Figure S8).

**Figure S8. US of a metastatic lymph node with dynamics towards a decrease in the size of the node and an increase in the area of fibrosis.**

After 5 months from the start of observation, stabilization of the process in terms of size and changes in the echostructure was noted (Figure S9). Throughout this period, according to the CDC, pronounced intranodular neovascularization persisted.

**Figure S9. US of a metastatic lymph node after the first 5 month.**

At the next visit, the echostructure of the node remained unchanged; its dimensions were 3.16×2.37×3.09 cm. However, nearby, more medially, and visually separately, a newly detected lymph node with preserved cortical-medullary differentiation of 6.8×4.9 mm, cortical layer up to 0.14 cm was registered (Figure 10). Below it, in close proximity to the target lymph node, a hypoechoic heterogeneous lymph node with fuzzy, uneven edges measuring 1.53×0.69 cm was identified (Figure S11).

**Figure S10. US of a metastatic lymph node with the newly visualized lymph node nearby (1).**

**Figure S11. US of a metastatic lymph node with the newly visualized lymph node nearby with** **hypoechoic heterogeneous echostructure.**

In the dynamics, an increase in the size of the lymph nodes detected for the first time during the previous scan was observed to 0.74×0.47 cm and 1.76×0.85 cm (Figure S12).

**Figure S12. US of a metastatic lymph nodes with dynamics to increase in size.**

Eight months after the start of observation were characterized by positive dynamics in the size of all registered neoplasms (Figure S13).

**Figure S13. US of a metastatic lymph node after the first 8 month.**

The marker foci were recorded in the dimensions of 2.86×2.25×2.64 cm, the echostructure and CDC data remained unchanged. The lymph node with preserved cortical-medullary differentiation was 0.78×0.47 cm, the cortical layer was unchanged, and the heterogeneous lymph node decreased to 1.13×0.95 cm.

During the last control examination, an increase in the size of lymph nodes with a heterogeneous structure from the previous examination was noted: 3.2×2.28×2.79 cm, 1.49×0.89 cm and a decrease in the size of the lymph node with preserved cortical-medullary differentiation, 0.44×0.41 cm (Figure S14). However, the size of the target lymph node did not exceed the values recorded at initial presentation.

**Figure S14. Final US of a metastatic lymph nodes with dynamics to increase in size.**

CT reports:

During dynamic radiological observation, a change in the linear dimensions of the lymph node under study was noted according to computed tomography data. In particular, compared to the previous CT scan from 01.31.2024, in which the dimensions of the lymph node were 34×24 mm, the CT scan from 10.10.2024 revealed a progressive increase in its dimensions to 44×38 mm, which indicated a negative dynamics of the process. At the same time, in the period from 10.10.2024 to 03.04.2025, according to the results of control CT scans, a tendency to decrease the linear dimensions of the lymph node to 35×26 mm was recorded, which can be regarded as positive dynamics against the background of the treatment (Figures S15-17).

.

**Figure S15. 03.04.2025**

**Figure S16. 25.04.2025**

**Figure S17. 04.09.2025**
